# The impact of sphingosine kinase inhibitor-loaded nanoparticles on bioelectrical and biomechanical properties of cancer cells

**DOI:** 10.1039/c5lc01201e

**Published:** 2015-11-26

**Authors:** Hesam Babahosseini, Vaishnavi Srinivasaraghavan, Zongmin Zhao, Frank Gillam, Elizabeth Childress, Jeannine S. Strobl, Webster L. Santos, Chenming Zhang, Masoud Agah

**Affiliations:** a Department of Mechanical Engineering , Virginia Tech , Blacksburg , VA 24061 , USA; b The Bradley Department of Electrical and Computer Engineering , Virginia Tech , Blacksburg , VA 24061 , USA . Email: agah@vt.edu; c Department of Biological Systems Engineering , Virginia Tech , Blacksburg , VA 24061 , USA . Email: chzhang2@vt.edu; d Department of Chemistry , Virginia Tech , Blacksburg , VA 24061 , USA

## Abstract

Cancer progression and physiological changes within the cells are accompanied by alterations in the biophysical properties. Therefore, the cell biophysical properties can serve as promising markers for cancer detection and physiological activities. To aid in the investigation of the biophysical markers of cells, a microfluidic chip has been developed which consists of a constriction channel and embedded microelectrodes. Single-cell impedance magnitudes at four frequencies and entry and travel times are measured simultaneously during their transit through the constriction channel. This microchip provides a high-throughput, label-free, automated assay to identify biophysical signatures of malignant cells and monitor the therapeutic efficacy of drugs. Here, we monitored the dynamic cellular biophysical properties in response to sphingosine kinase inhibitors (SphKIs), and compared the effectiveness of drug delivery using poly lactic-co-glycolic acid (PLGA) nanoparticles (NPs) loaded with SphKIs *versus* conventional delivery. Cells treated with SphKIs showed significantly higher impedance magnitudes at all four frequencies. The bioelectrical parameters extracted using a model also revealed that the highly aggressive breast cells treated with SphKIs shifted electrically towards that of a less malignant phenotype; SphKI-treated cells exhibited an increase in cell-channel interface resistance and a significant decrease in specific membrane capacitance. Furthermore, SphKI-treated cells became slightly more deformable as measured by a decrease in their channel entry and travel times. We observed no significant difference in the bioelectrical changes produced by SphKI delivered conventionally or with NPs. However, NPs-packaged delivery of SphKI decreased the cell deformability. In summary, this study showed that while the bioelectrical properties of the cells were dominantly affected by SphKIs, the biomechanical properties were mainly changed by the NPs.

## Abstract 2

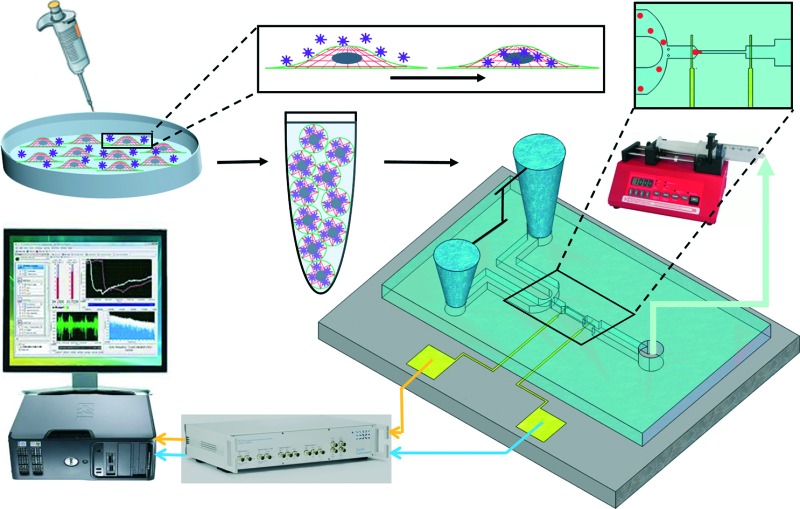
A microfluidic chip developed to study the effects of free-drug *versus* NPs-mediated drug delivery on cancer cells using their electromechanical biomarkers.

## 1. Introduction

The biophysical properties of cells including their biomechanical and bioelectrical properties vary as a function of their tumorigenicity, metastatic potential, and health state. A more thorough understanding of cancer pathology, with possible gains in therapeutic insights, might be achieved through development of methods to monitor how cancer comes to dysregulate cell biophysical behaviors.^1^ Cancer pathology directly impacts and dysregulates cell biophysical behaviors through changes in cell membrane, cytoskeleton, and cytosol composition. The decrease in the cell stiffness and viscosity is a well-documented biomechanical signature during cancer progression which facilitates metastasis.^2–4^ This change in the cell biomechanical properties is associated with the disorganization and decrease in concentration of the fundamental components of the cell cytoskeleton.^5^ Furthermore, bioelectrical properties of cells are also altered during cancer progression because of the changes in cell membrane composition and internal conductivities.^6,7^ The use of bioimpedance analyzers has gained broad acceptance for cancer metastatic diagnosis at single-cell resolution.^8,9^ In this regard, cancer chemotherapeutic agents are purposely designed to target the cell structure, and consequently alter cell biophysical characteristics. The effects of drugs on biophysical properties of cells have been evaluated to provide insights into the sensitivity and efficiency of chemotherapies.^10–14^

However, chemotherapy is often non-specific to cancer cells, which causes many severe toxic side-effects. In contrast to the conventional method of delivering medications, nanoparticles (NPs) offer new approaches to drug-packaged delivery as a means to reduce off-target toxicity and enhance drug bioavailability by improving the timed release of drugs.^15,16^ NPs are being used for targeted drug-delivery to cancer cells.^17,18^ It is notable that while the delivery of anti-cancer drugs to the specific cells can provide the desired chemotherapeutic effects, the side-effects of intracellular NPs are often unclear. Several studies have analyzed the changes in the biomechanical properties of cells and their cytoskeleton architecture when exposed to NPs.^19,20^ These studies utilizing atomic force microscopy are mainly focused on adhered cells. For instance, the recent results indicate that the stiffness of mesenchymal stem cells increased under the impact of silica (Si) and silica-boron (SiB) NPs as a result of F-actin structural reorganization.^21^ Moreover, hematite NP-treated *Escherichia coli* cells become significantly stiffer than untreated cells.^22^ In addition, the super-paramagnetic iron oxide NPs increased cell elastic modulus of endothelial cells by 50% and formed actin stress fibers within the cells.^20^ However, there are some other studies with opposing results on cell biomechanics. For example, selenium (Se) NPs have been shown to remarkably decrease the Young's modulus of MCF-7 cells by disturbing membrane molecules and F-actin and inducing toxicity.^23^ All these observations indicate that NPs have significant impact on cell structure and so the biophysical attributes. The combinatory effects of NPs and chemotherapeutic agents on cancer cells by means of the biophysical markers is untouched despite its significance.

This study aims to investigate the impact of new potential anti-cancer drugs,^24^ sphingosine kinase inhibitors (SphKIs), delivered by NPs on cancer cells utilizing a single cell-based assay. Human cancer tissues elevate sphingosine kinase (isoforms: SphK1 and SphK2), which results in increased production of sphingosine-1-phosphate (S1P) from sphingosine. These sphingolipid metabolites are involved in diverse cellular processes^25^ as well as cancer pathogenesis and treatment.^26^ We have previously determined their differential effects on the biomechanical properties of cells as they transition to cancer.^27^ S1P is a ubiquitous signaling molecule that acts as a ligand for five G-protein coupled receptors (S1P1-5) whose downstream effects are implicated in a variety of important pathologies including cancer, inflammation, and fibrosis. S1P is an important molecule that controls vascular barrier function, vascular tone, and regulation of lymphocyte trafficking by acting through S1P receptors. The ability of S1P (hence, SphK) to alter the permeability of vascular system is important in cancer metastasis. The synthesis of S1P is catalyzed by SphK and hence, inhibitors of this phosphorylation step are pivotal in not only understanding but also in halting the metastatic transition of cancer cells.

Microfluidic technology has emerged as a potential high-throughput technique for determining biophysical signatures at single-cell resolution.^28–33^ The significance of single-cell resolution assessment is further highlighted by considering that many biological experiments are carried out on cell populations ignoring the fact cancer masses are comprised of a heterogeneous mixture of cancer cells.^34^ In this study, a high-throughput, label-free microfluidic chip is developed for screening biophysical (bioelectrical and biomechanical) properties of individual cells in heterogeneous cell populations. The uniquely designed microfluidic chip equipped with embedded parallel microelectrodes enables the deformation of single cells as they pass through a constriction and fully automated single-cell bioelectrical (multi-frequency impedance magnitudes) and biomechanical (entry and travel times) measurements. In this microchip, impedance is continuously monitored in real-time as cells transition between a mechanically non-disruptive channel into a narrow deformation region producing mechanical stress by deforming the cell membrane, cytoplasm, and nuclear structures, and as cells relax upon exiting the deformation region. This microchip is sensitive to alterations in cell biophysical properties and has application to detection of cellular responses to pharmaceutics.

To evaluate NP-mediated drug delivery, SphKIs were loaded into biodegradable poly lactic-co-glycolic acid (PLGA) NPs.^35,36^ Human MDA-MB-231 epithelial cells representative of highly invasive breast cancer were exposed to SphKIs treatments, then introduced into the microfluidic device where biophysical measurements were captured and compared. Concurrent biological experiments and mathematical modeling were carried out as an approach to associate biophysical alterations with cell structural components. The selective targeting of SphKIs to cancer cells using NPs coupled to determining cellular structural changes using a single-cell resolution microfluidic chip is novel. The approaches taken in this work can be applied to the analysis of NPs carrier-effects on cells as well as to drug screening and development of new cancer drugs to deter cancer progression by reversing aberrant biophysical properties.

## 2. Materials and methods

### 2.1 Sample preparation

MDA-MB-231 human epithelial breast cancer cell line (ATCC; American Type Culture Collection, Manassas, VA) representing highly invasive breast carcinoma was chosen in this study. MDA-MB-231 cells were grown in Dulbecco's modified Eagle's medium (DMEM) (ATCC, Manassas, VA) containing 10% fetal bovine serum (Atlanta Biologicals, Norcross, GA), penicillin-streptomycin (100 Units per ml), and 4 mM l-glutamine. The cells were grown in T-25 cm^2^ culture flasks at 37 °C in humidified 5% CO_2_–95% air atmosphere. For free-drug treatment of cancer cells with the SphKIs, each compound was added to the cell culture medium at a non-toxic concentration of 10 μM for the specified times indicated in the results section. For NP-packaged treatment of cancer cells, the PLGA NPs were introduced into the cell culture medium at a 10 μM final concentration of each SphKI loaded in NPs for the specified periods. For experiments, cells were harvested from confluent cell culture flasks and suspended (5 × 10^5^ cells per mL) in the growth medium.

### 2.2 Western blot analysis

To quantify the relative content of actin proteins before and after treatments, Western blot (WB) analysis was performed. The cells were cultured to 70% confluency in a culture flask, and then harvested in a modified radio immunoprecipitation assay (RIPA) buffer (10 mM Tris-HCl, 1 mM EDTA, 1% Triton X-100, 0.1% sodium deoxycholate, 0.1% SDS, 140 mM NaCl) to obtain a whole-cell lysate. Samples were loaded onto a 4–12% SDS polyacrylamide gel for electrophoresis. A WB was performed with a Biorad Transblot Turbo system (Bio-Rad Laboratories Inc., Hercules, CA). Blocking of the membrane was done with Tween Tris buffered saline (TTBS) containing 5% nonfat dry milk. After two washes, the membrane was incubated with primary antibody solution containing a 1 : 2000 dilution of mouse anti–actin (Sigma Aldrich, St. Louis, MO) in blocking buffer. Two washes in TTBS were performed and the membrane was incubated with a secondary antibody solution containing a 1 : 2000 dilution of goat anti-mouse IgG antibody (Sigma Aldrich, St. Louis, MO) coupled to horseradish peroxidase (HRP). Chemiluminescence with Biorad Clarity substrate (Bio-Rad Laboratories Inc., Hercules, CA) was used to detect the presence of antibodies. Densitometry was performed using Biorad Image Lab software.

### 2.3 Immunofluorescence imaging

For confocal microscopy, cells were grown on culture plates for 24 h before treatments; 48 h after treatments, the cells were washed in Hank's balanced salt solution (HBSS), fixed with 3% paraformaldehyde (PF) in 250 mM Tris, pH 7.2 for 10 minutes followed by 6% PF–0.25% Triton X-100 in phosphate-buffered saline (PBS) for 10 minutes. The PLGA NPs were fluorescently labeled in their synthesis process by adding 0.2 mg mL^–1^ Nile red. For actin cytoskeleton staining, the cells were incubated with Alexa Fluor-488 phalloidin (Invitrogen, Carlsbad, CA) at room temperature (5 U ml^–1^ in 140 mM NaCl–6% bovine serum albumin in 40 mM Tris, pH 7.2, Invitrogen) for 15 minutes. Then, the samples were rinsed three times in PBS and mounted on ProLong Gold antifade reagent with DAPI (Invitrogen, Carlsbad, CA) to stain the cell nuclei. The confocal imaging was performed on the samples using a confocal laser scanning microscope (LSM510, Zeiss, Thornwood, NY).

### 2.4 TEM imaging

Transmission electron microscopy (TEM) was performed to investigate the distribution of the NPs in the cells. For TEM imaging, the cells were grown on culture plates for 24 h before adding the NPs. After 24 h incubation, the culture medium was removed and the cells were washed 2 times with 0.1 M Na-cacodylate for 15 minutes, and then post-fixed with 1% OsO4 in 0.1 M Na-cacodylate buffer, pH 7.2 for at least 1 h. The buffer was removed and the cells were washed 2 times again in 0.1 M Na-cacodylate for 10 minutes. Afterward, the cells were dehydrated using an ascending ethanol series ending in 100% ethanol (15 minutes in each of five ethanol solutions), and then in propylene oxide for 15 minutes. After dehydration, the cells were infiltrated with a 50 : 50 solution of propylene oxide : Poly/Bed 812 for 6–24 h. Then, the cells were embedded in 100% Poly/Bed 812, and placed in 60 °C oven for at least 48 h to cure. Finally, the samples were cut to 90–150 nm thick sections for TEM imaging. The images were acquired using a JEOL JEM 1400 TEM (JEOL Ltd., Tokyo, Japan).

### 2.5 Sphingosine kinase inhibitors

The most potent and selective SphKIs including SphKI1 and SphKI2 as well as a dual inhibitor, DuaLI were discovered and synthesized by Santos's group.^37–39^ SphKIs were dissolved in dimethyl sulfoxide (DMSO) solvent before use as a concentrated stock solution at 4 °C which was diluted into cell culture medium for conventional free-drug delivery. Alternatively, the SphKIs were incorporated into NPs as detailed below.

### 2.6 Nanoparticles

Drug-loaded biodegradable PLGA NPs were fabricated by fluidic nanoprecipitation method.^35^ In brief, 1 ml DMSO/acetone (1 : 9 v/v) was used to dissolve 25 mg PLGA and 5 mg of each SphKI which was then injected into 5 ml 0.5% polyvinyl alcohol (PVA) solution perpendicularly under continuous stirring (1200 rpm). The resulting suspension was stirred overnight to allow complete acetone evaporation. NPs were collected by centrifugation for 30 min at 10 000*g*, and were washed three times using ultrapure water. NPs without drugs were prepared using the same method. Drug loading efficiency was determined by disrupting NPs using 1 M NaOH followed by high-performance liquid chromatography (HPLC) analysis. Drug release was detected by dialyzing 8 mg drug loaded NPs against 50 mL 10 mM PBS buffer (pH 7.4) using a dialysis tube with molecular weight cut-off (MWCO) of 6000–8000 daltons. At predetermined time points, 3 mL of dialysate was taken out and replaced with equal volume of fresh buffer.

### 2.7 Microfluidic chip

**Fig. 1:**
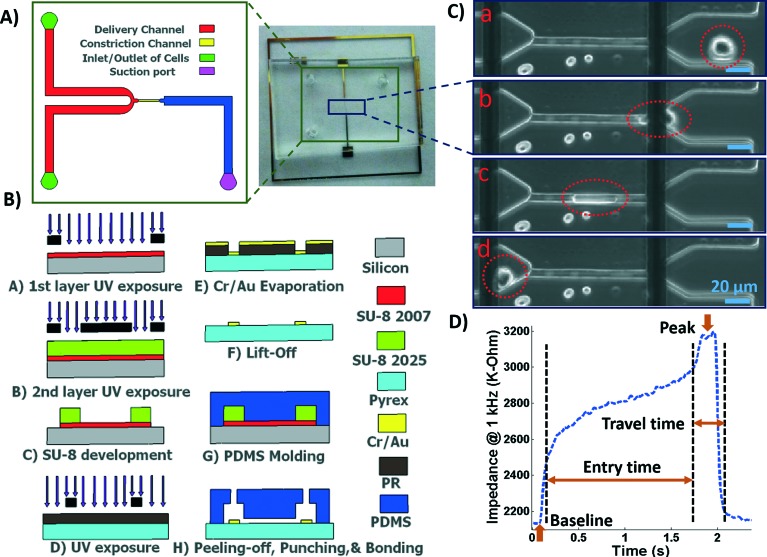
A) Illustration of the fabricated microfluidic chip including delivery and constriction channels, cells inlet/outlet, and suction port. B) Process flow for fabrication of the microfluidic device. C) Image showing a single deformed cell travelling through the constriction channel. D) The impedance magnitude change at 1 kHz when a single cell passes through the constriction channel.

A microfluidic chip was designed and fabricated for high-throughput biophysical profiling of single cells. The two principal parts of the microchip are the constriction and delivery channels that deliver, trap, and pass the cells continuously as shown in Fig. 1A. The U-shaped delivery channel is a mechanism to deliver single cells at the entrance of the constriction channel to prevent clogging. A continuous free flow of undeformed suspended cells in culture medium is established in the delivery channel between the inlet/outlet by a difference in the level of solution in the reservoirs. The constriction channel dimensions are designed to be narrow (8 μm-wide), shallow (8 μm-deep), and straight (100 μm-long) to enable deformation of cells as they are pulled through the constriction microchannel. Single cells are trapped and pulled continuously through the constriction channel as a result of constant pressure of –150 Pa imposed by a syringe pump (Harvard Apparatus, Holliston, MA) connected to another end of the constriction channel. Once a cell is trapped and is traveling through the microchannel, it completely blocks the constriction channel so that another cell never enters. A parallel microelectrode pair is integrated on either side of the constriction channel for simultaneous measurements of the impedance at multiple frequencies and entry and transit times of single cells automatically as they pass through the constriction channel.

The polydimethylsiloxane (PDMS) layer was obtained by casting on a two-layer SU-8 (MicroChem Corp., Westborough, MA) master fabricated using standard soft lithography techniques. Two layers of SU-8 on a silicon wafer are to obtain the shallow constriction channel (8 μm) and the deep delivery channel (30 μm) as shown in Fig. 1A. The process flow for fabrication of the microchip is shown in Fig. 1B. Briefly, the first layer of SU-8 (8 μm thick, SU-8 2007) was made to form the constriction channel, which was spun on the wafer, soft-baked, and exposed to UV light through the first chrome-on-glass mask. The wafer was then baked on a hot plate to cross-link the exposed SU-8. The second layer of SU-8 was made to form the delivery channel, which (22 μm thick, SU-8 2025) was spin-coated on the wafer, soft-baked, aligned, and then exposed to ultraviolet (UV) light through the second film mask, followed by post-exposure bake. Finally, the two-layer SU-8 was developed and hard baked. PDMS pre-polymer was mixed with the curing agent at 10 : 1 ratio and molded onto the SU-8 master placed in an aluminum foil plate. The wafer was then placed in a vacuum desiccator to degas air bubbles in the PDMS before curing on a hot plate. The PDMS device was allowed to cool and then was peeled from the SU-8 master and diced. Inlet/outlet holes and suction port were punched into the PDMS.

To fabricate the electrode layer, photoresist AZ9260 was first spun coated on the Pyrex/glass wafer. After exposure through a mask and development in AZ400k, the electrode pair pattern with a minimum width of 20 μm and spacing distance of 120 μm was transferred onto the Pyrex/glass wafer. Then, a 25 nm/100 nm layer of Cr/Au was deposited on the wafer by evaporation (PVD, Kurt J. Lesker). Following by a lift-off process in acetone, the electrodes were patterned in the areas that were unprotected by the photoresist. The Pyrex wafer was then diced using the MA-1006 dicing saw to yield individual electrode chips. The electrode and the PDMS layers were exposed to oxygen plasma cleaner (Harrick Plasma, Plasma Cleaner); the electrodes on the Pyrex chip were aligned with the constriction channel under a microscope using a few drops of methanol, pressed together and transferred onto a hot plate to bond.

### 2.8 Data acquisition and analysis

The microchip was mounted on a general purpose board (GPB) with subminiature version A (SMA) adaptors. The HF2IS impedance spectroscope (Zurich instruments, Zurich, Switzerland) was used for continuous impedance measurements as a measure of the opposition to the flow of electric current. The impedances were measured at four frequencies of 1 kHz, 10 kHz, 100 kHz and 1 MHz in parallel using an excitation voltage of 2.25 V at each frequency. The Redlake NX-3 high speed camera (IDT, Pasadena, CA) was used to monitor and image the cells at the rate of 500 fps. Fig. 1C shows tracking of a cell in the captured images as it passes through the constriction channel. Fig. 1D shows the characteristic impedance profile at 1 kHz, which corresponds to the impedance changes recorded as the single cell moves through the constriction channel. The impedance magnitude measured from the culture medium alone is the baseline value. When a cell approaches the entrance of the constriction channel (Fig. 1C-a), the impedance increases with a steep slope. As the cell is trapped and squeezed into the constriction channel (Fig. 1C-b), the impedance magnitude rises gradually. The time the cell takes to deform and squeeze into the constriction channel is called the entry time. When the cell completely enters and is at the center of the microchannel, the impedance magnitude suddenly rises and reaches a peak value (Fig. 1C-c). The cell exits the constriction channel rapidly as can be seen from the impedance's steep slope back to the baseline (Fig. 1C-d). The time the cell spends traveling through the constriction channel is called the travel time. Therefore, the entry and travel times of the cell through the constriction microchannel can be calculated from the impedance profile.^40^ The impedance change between the peak and the baseline for single cells was calculated using real and imaginary parts of their complex impedance values. After data acquisition, MATLAB program was used for rapid analysis of the cell entry and travel time through the microchannel, and identifying the magnitude of impedance change at each frequency for every cell. The curve fitting for extracting cell bioelectrical parameter values were performed in MATLAB program using the nonlinear least squares method (*R* ^2^ > 0.95). The experiments were conducted for populations with >100 cells from each sample. The throughput of the microchip was as high as 20 cells per min. This throughput can be further increased by changing the negative pressure or by integrating parallel constriction channels. To examine and validate the robustness and reproducibility of the developed microchips and their stability over time, at least three separate tests were conducted for each cell population using one chip over time and/or different chips. Within the same cell population, there was no more than 5% variation between the average measured parameters of any two tests. As a note, this microchip is quite versatile and can be used to make measurements from single cell suspensions of mammalian cells including other cancer cell types. *P*-values between the different populations were calculated using two independent samples *t*-tests (*α* = 0.05). Results in graphs are presented as arithmetic mean ± standard error of the mean (SEM). All statistical tests were performed using GraphPad Prism software.

## 3. Results and discussion

**Fig. 2:**
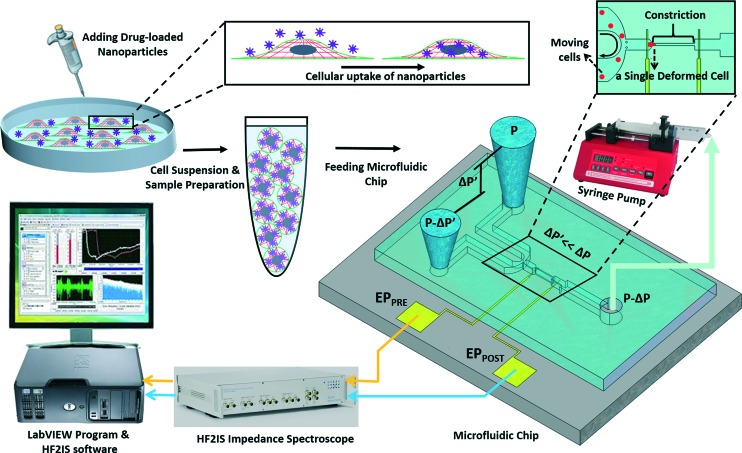
Schematic image showing the operation of the microfluidic chip.

The experimental set-up and operation of the microchip is shown schematically in Fig. 2. Indeed, as an unprecedented microfluidic-based study, the effects of potential anticancer agents, SphKIs in conventional free-drug *versus* NP-packaged drug delivery on the highly metastatic cells are explored by means of the biophysical markers. The microfluidic chip includes an electrode pair embedded on either sides of a narrow constriction channel which serves the dual purpose of automating the entry and travel time measurements and enabling multi-frequency impedance measurements simultaneously as single cells pass continuously through the constriction channel. The HF2IS impedance spectroscope connected to the microchip continuously records impedance signals in communication with LabVIEW program and HF2IS software on computer.

### 3.1 Nanoparticles characterization

**Fig. 3:**
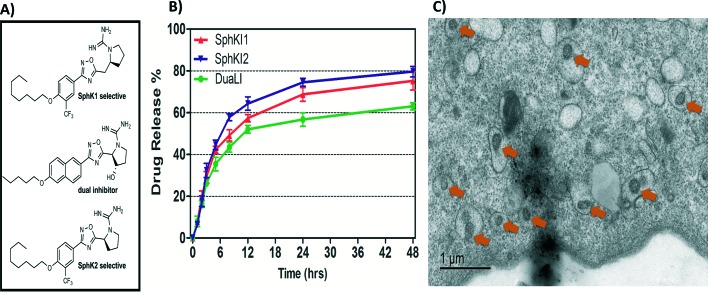
A) Formula of the drugs loaded in NPs. B) Release profiles of drug-loaded NPs. C) TEM image of NPs uptake in one cell.

Biodegradable and biocompatible PLGA NPs loaded with SphKIs are characterized before the application. Fig. 3A shows the chemical structure of the NP encapsulated SphKI compounds. In Fig. 3B, the drug release profiles of drug from the NPs as determined by dialysis against a physiological buffer are shown. What we see is that all three drugs exhibit similar release profiles, with a 50% drug release of approximately 8 hours. Also, drug release profiles do not reach 100%. It is because during the *in vitro* release study, PLGA NPs maintain their structure. Therefore, the tight encapsulation and interactions between drug molecules and NPs will keep some drugs unreleased in the study period. Subsequently, the dimension and the distribution of the NPs after accommodation in cells were monitored by the TEM image as shown in Fig. 3C. The NPs have a uniform size and spherical shape with 150 nm mean diameter.

### 3.2 Cell bioelectrical characterization

**Fig. 4:**
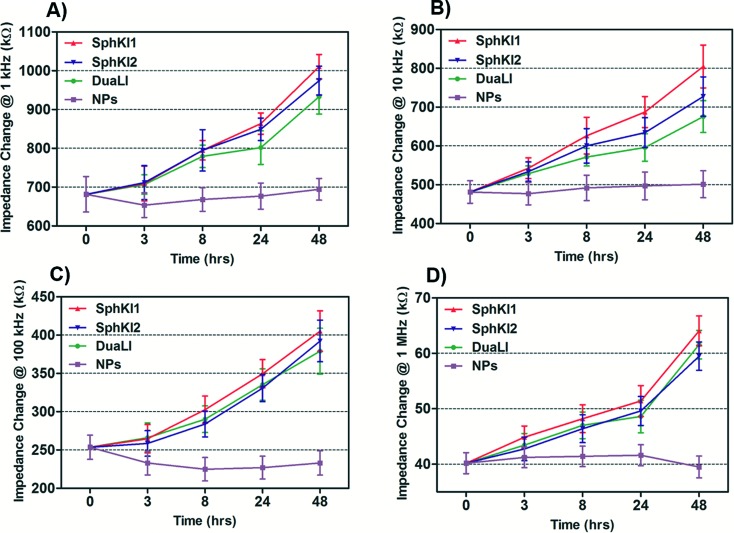
Measured impedance changes at A) 1 kHz, B) 10 kHz, C) 100 kHz, and D) 1 MHz frequency recorded as single MDA-MB-231 cells after treatments pass through the constriction channel.

The impedance data were measured simultaneously at the four frequencies as single cells move through the constriction channel. Fig. 4(A–D) shows the mean ± SEM of the maximum changes in the impedance magnitude occurring at different time lapses after treatments at each frequency. There was a significant increase in the mean impedance magnitude of the MDA-MB-231 cells after conventional free-drug delivery of either of the three SphKIs. The increase was more pronounced at higher frequencies. In comparison, no significant differences in the mean values of the impedance magnitude occurred in the cells treated with unloaded NPs. Thus, the presence of NPs inside the cells did not appreciably change their bioimpedance characteristics. The mean impedance magnitudes of the MDA-MB-231 cells after exposure to NP-packaged SphKIs (data not shown) were similar to those seen for the conventional (free-drug) delivery at all frequencies. It was previously observed that that the impedance magnitude of the tumorigenic cells was on average significantly lower than that of non-tumorigenic cells;^41^ the results here, showed that the SphKIs raise the impedance magnitude of the highly aggressive breast MDA-MB-231 cells to values more typical of a less tumorigenic phenotype.

**Fig. 5:**
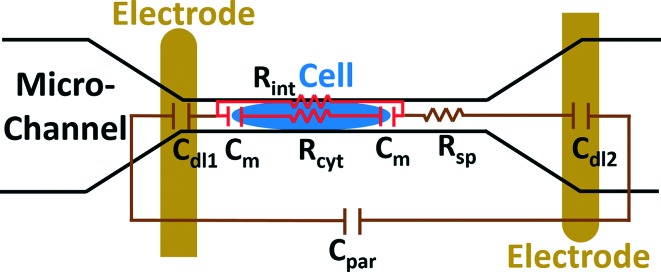
A schematic illustration of the electric circuit model for the microchannel with an elongated cell used to characterize cell electrical parameters from the multi-frequency impedance measurements. Circuit element legend: *C*_dl1_ and *C*_dl2_ – double layer capacitance; *C*_par_ – parasitic capacitance; *C*_m_ – membrane capacitance; *R*_sp_ – spreading resistance; *R*_cyt_ – cytoplasm resistance; *R*_int_ – cell-channel wall interface resistance.

The electric circuit model for the constriction microchannel with an elongated cell shown in Fig. 5 is used to extract cell bioelectrical parameters.^42,43^ The baseline impedance occurs when no cells are present near the electrodes and the peak impedance occurs when a cell is located in the middle of constriction channel. The electric circuit used to model the microchannel consists of the capacitance of the double layers formed at the interface of the electrodes (*C*_dl1_, *C*_dl2_) in series with the spreading resistance (*R*_sp_) of the culture medium in the constriction channel. A parallel parasitic capacitance (*C*_par_) is also considered in the microchannel circuit model. For the living cell, the cell membrane can be modeled as a capacitance (*C*_m_) and the cell cytoplasm as a resistance (*R*_cyt_).^42,43^ A leakage resistance is also present in the path of current flow in the interface of the cell and the wall of the constriction channel which is represented by the interface resistance (*R*_int_). As a note, all capacitors in this electrochemical circuit are generally modeled as constant phase elements. *C*_dl_, *C*_par_ and *R*_sp_ were obtained initially using the baseline impedance. They were later used to obtain *C*_m_, *R*_cyt_ and *R*_int_ using the peak impedance. The specific membrane capacitance was obtained by dividing the membrane capacitance by surface area of cell head and tail in the constriction channel which are estimated as the hemispherical surface area (4π*r* ^2^ where *r* = 4 μm). The cytoplasm conductivity was obtained from *l*/(*R*_cyt_·*A*) where *l* is the cell length in the channel and *A* is the channel cross section area (8 × 8 μm^2^).

**Fig. 6:**
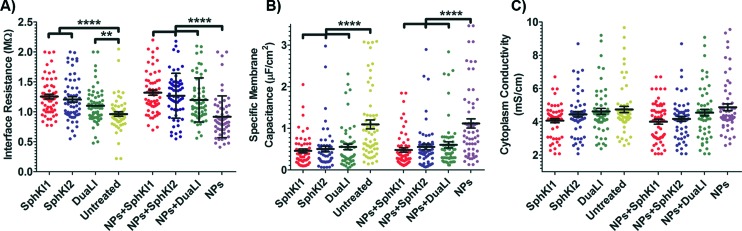
Scatter plots of (A) interface resistance, (B) specific membrane capacitance, and (C) cytoplasm conductivity obtained for 48 h treated cells with free-drug and NP-packaged SphKIs in comparison to untreated and unloaded NPs-treated cells, respectively. **P ≤ 0.01, ****P ≤ 0.0001.

The model presented in Fig. 5 was fitted to the data from the multi-frequency impedance measurements to extract the cell bioelectrical parameters. The interface resistance, specific membrane capacitance, and cytoplasm conductivity of the MDA-MB-231 cells after a 48 h treatment with free and NP-packaged SphKIs in comparison to untreated and unloaded NP-treated cells, respectively, are extracted and depicted in Fig. 6(A–C). According to Fig. 6A, interface resistance (*R*_int_) is significantly larger after both conventional free-drug and NP-packaged delivery of SphKI1, SphKI2, and DuaLI. Thus, treatment of the breast cancer cells with SphKIs increased the resistance at the interface between the cell and the channel wall, which might reflect increased surface friction. Our previous results also showed that non-tumorigenic cells have significantly higher *R*_int_ values compared to the highly metastatic cells (unpublished data). In addition, as shown in Fig. 6B, the cells treated with one of SphKIs, regardless of delivery method exhibited on average a significantly lower specific membrane capacitance. The membrane capacitance can be changed due to alterations in lipid composition, surface charges, and ion channel regulation.^44^ The membrane of tumorigenic cell is found relatively enriched in many kinds of lipids,^45^ which causes tumorigenic cells to show an increased membrane capacitance.^46,47^ In fact, as a result of SphKIs treatment, the bioactive sphingolipid metabolite, S1P is expected to be diminished. Previously published results showed that S1P increases cell membrane capacitance.^48^ Since SphK catalyses formation of S1P, hence inhibition of SphK might explain reversal of the membrane capacitance of the breast cancer cells following treatments with the three SphKIs. The cytoplasm conductivity is another extracted parameter reflecting the cell interior's bioelectrical properties. Notably, the cytoplasmic conductivity of the breast cancer cells did not considerably change after SphKIs treatment. This suggests that either the possible reorganization of internal cytoskeleton by SphKIs or the presence of NPs in cell's cytoplasm did not cause significant changes in the cell's bioelectrical conductivity. Taken together, the reported bioelectrical parameters of the MDA-MB-231 cells after treatment with SphKIs indicate a modification in the interface resistance and the membrane capacitance of the highly aggressive breast cells.

### 3.3 Cell biomechanical characterization

**Fig. 7:**
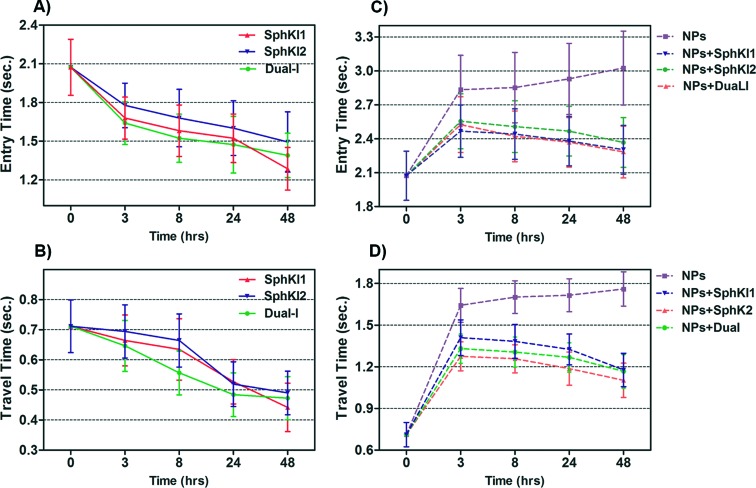
Measured entry time and travel time changes recorded as single MDA-MB-231 cells before and after treatments with free-drug (in A and B) and NP-packaged SphKIs (in C and D) pass through the constriction channel.

Total transit time of the cells through the constriction channel can be broken down to entry and travel times. The entry and travel time stamps can provide information about cell biomechanical properties.^49,50^ The entry time is that in which the cells gradually deform to enter into the constriction channel; entry time is related to cell viscosity. The travel time is that in which the cells maintain a nearly constant shape and speed is related to cell stiffness.^51^ Fig. 7(A and B) and Fig. 7(C and D) show the entry and travel times of cells at different time lapses after conventional free-drug and NP-packaged drug delivery, respectively; impedance data was used to track entry and travel times. The entry and travel times through the constriction channel follow a similar pattern for different samples. Fig. 7A shows that the entry times for MDA-MB-231 cells population continuously deceased by time after free-drug treatments with the three SphKIs. These measurements indicate that the mean ± SEM of entry times for MDA-MB-468 cells (2.072 ± 0.217 s) became statistically shorter (*P* < 0.01) after 48 h free-drug treatments with SphKI1 (1.285 ± 0.165 s), SphKI2 (1.493 ± 0.233 s), and DuaLI (1.389 ± 0.172 s). Also, according to Fig. 7B, the average travel time of MDA-MB-231 cells through the microchannel was 0.711 ± 0.087 s which decreased to 0.441 ± 0.080 s, 0.490 ± 0.072 s, 0.472 ± 0.071 s (*P* < 0.05) after free-drug treatments with SphKI1, SphKI2, DuaLI, respectively. Some previously published AFM-based stiffness measurements also reported that S1P can increase the stiffness of cells to some extent.^52,53^ Since SphK catalyses the formation of S1P, inhibitors of SphK can have opposite effects on the cell stiffness which justifies the decrease in cell deformability as a result of treatment with the three SphKIs. In contrast, according to Fig. 7C and D, the cells showed longer entry and travel times after just 3 h treatment with unloaded NPs, although this increasing trend was lower after the initial 3 h probably because the NPs' internalization rate decreases. This 3 h time is enough for the NPs to be up taken by the cells. The increase in the stiffness of different cells under the impact of NPs was previously shown.^20–22^ Also, after NP-packaged SphKIs treatments of MDA-MB-231 cells, there was an initial increase in the entry and travel times followed by a decrease in the convening hours when the SphKIs are released. The initial rise in the times is due to the absorbance of NPs while the following decreases are apparently because the effects of the released drugs dominated the effects of internalized NPs.

To investigate if the effects of NPs on the cell biophysical properties are dependent on NPs size and/or composition, we tested MDA-MB-231 cells treated with unloaded PLGA NPs having mean diameter of 50 μm and 300 μm; we also examined effects of liposomal NPs. The results confirmed that the NPs at least within the tested type and size range have minimal effects on the measured cell bioimpedance. On the other hand, the cell entry/travel time measurements are dependent on NPs' size and type.

**Fig. 8:**
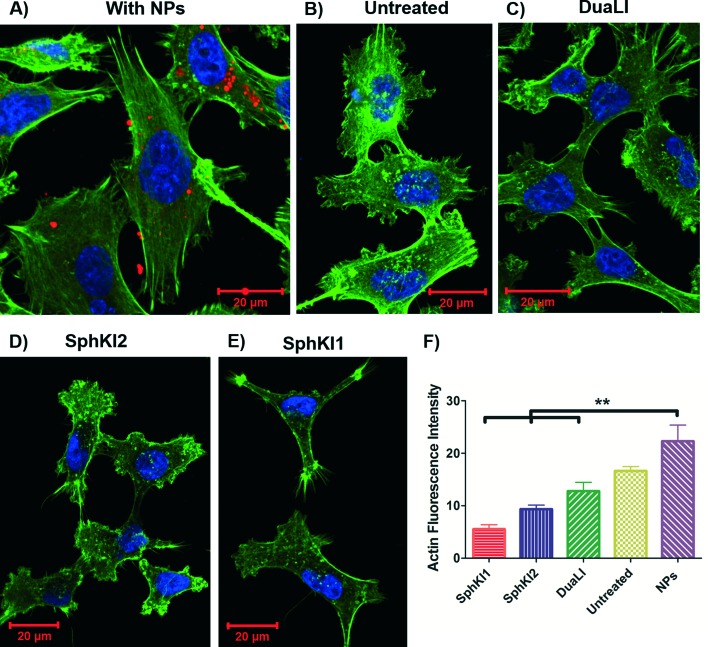
Immunofluorescence images showing difference in the actin organization of A) unloaded NP-treated, B) untreated, C) DuaLI treated, D) SphKI2 treated, and E) SphKI1 treated cells. F) Actin content intensity revealed treatments with SphKI, SphKI2, and DuaLI led to a decrease in the actin intensity, while the absorbed NPs increased the actin intensity. **P ≤ 0.01.

Cell biomechanical changes are attributed to the reorganization of the cell cytoskeletal proteins where actin filaments have been found to play the dominant role.^54^ Actin filament structure organizations were monitored using immunofluorescent staining to correlate the changes in the cell deformability to the alterations in their intracellular cytoskeleton. Fig. 8(A–E) shows the actin filaments of the untreated cells compared to the cells after SphKIs and NPs treatments. Accordingly, while the presence of NPs considerably regulated actin fiber organization, SphKIs treatment deregulated them. Moreover, the actin filament relative intensity from fluorescence staining was measured to identify the contribution of these changes in biomechanical properties of cells. Actin content intensity was obtained from at least three fluorescence stained images using ImageJ software and the results are shown as the mean ± SD (*P* < 0.01) in Fig. 8F. The intensity decreased after SphKIs treatment. This value was 16.6 for the untreated aggressive MDA-MB-231 cells while reduced to 5.5, 9.3, and 12.8 after treatments with SphKI1, SphKI2, and DuaLI, respectively. Furthermore, the level of fluorescence intensity for the cells noticeably increased to 22.3 after unloaded NPs treatment. In other words, treatment with the SphKIs reduces the actin microfilament intensity by approximately 66%, 44%, and 23%, for SphKI1, SphKI2, and DuaLI, respectively, while the presence of NPs led to an increase in content of actin by 34%.

**Fig. 9:**
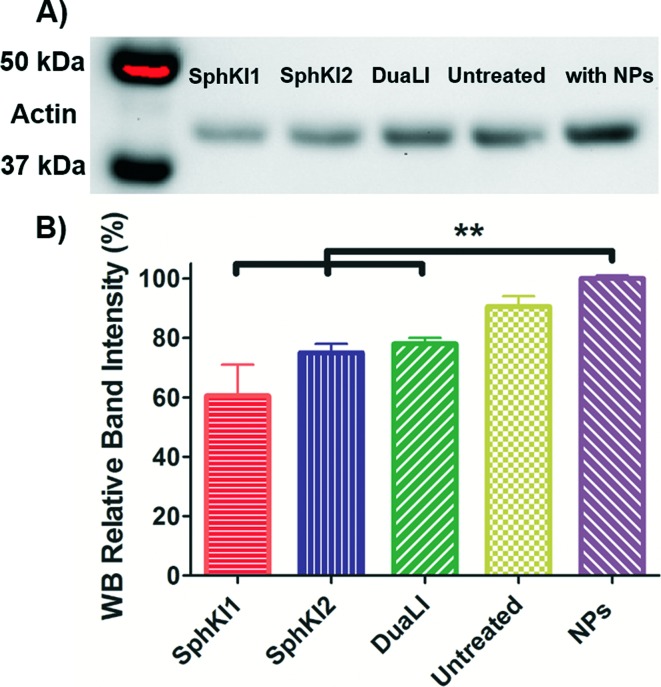
A) Actin filaments protein WB of whole-cell extracts from untreated cells and cells treated with SphKI1, SphKI2, DuaLI, and unloaded NPs for 48 h. B) Normalized (to NPs) levels of actin protein bands in treated and untreated cells. **P ≤ 0.01.

WB analysis was also performed to quantify the relative actin content of cells. Fig. 9A shows the results of WB analysis. Three biological replicates performed and the relative band intensities of the samples normalized to the largest one are shown as the mean ± SD (*P* < 0.01) in Fig. 9B. Accordingly, treatments with SphKI1, SphKI2, and DuaLI led to a decrease in the actin content by approximately 33%, 17% and 14%, respectively. In contrast, the level of actin was 11% higher in the cells treated with unloaded NPs compared to the untreated cells. It can be concluded that treatments with SphKIs decrease the level of actin filaments, while NPs increase this level.

## Conclusions

A microfluidic chip system for high-throughput, label-free, automated single-cell measurements of biomechanical and bioelectrical properties was used to evaluate the effects of SphKIs on metastatic breast tumor MDA-MB-231 cells after conventional or NP-mediated drug delivery. The extracted bioelectrical parameters showed that SphKIs, but not NPs, modified cell-channel interface resistance and specific membrane capacitance of the cells. In contrast, the biomechanical properties of the metastatic cells measured by the constriction channel entry and travel times decreased slightly after SphKI free-drug treatments, indicating cell softening. However, these biomechanical properties increased significantly following SphKIs NP-packaged treatments suggesting that NP-mediated delivery of SphKI might result in an overall more therapeutic alteration in both bioelectrical and biomechanical tumor cell properties. The NPs alone modulated the cell biomechanical characteristics, increasing cell viscosity and stiffness and cytoskeletal actin as determined by immunofluorescence and WB analysis. This work demonstrates how combining single-cell biophysical (bioelectrical and biomechanical) analyses with drug screening provides a promising strategy to screen therapeutic avenues and identify new genres of cancer therapeutics that possess capabilities to reverse biophysical traits that take place during cancer progression.
